# miRNA limits MAP kinase-mediated immunity: optimization of plant fitness

**DOI:** 10.1093/jxb/erx385

**Published:** 2017-12-16

**Authors:** Shengjun Li, Bin Yu

**Affiliations:** Center for Plant Science Innovation and School of Biological Sciences, University of Nebraska, USA

**Keywords:** Cotton, feedback regulation, *Fusarium oxysporum*, MAPK cascade, microRNAs

## Abstract

This article comments on:

Wang C, He X, Wang X, Zhang S, Guo X. 2017. ghr-miR5272a-mediated regulation of GhMKK6 gene transcription contributes to the immune response in cotton. Journal of Experimental Botany 68, 5895-5906.

**Plants often face growth and defense trade-offs when encountering biotic and abiotic stresses. In research addressing this balance in the resistance of cotton to phytopathogenic fungi, Wang *et al.* (2017) have found that a riboregulator, ghr-miR5272a, limits excess immunity through inhibiting the expression of *MAPKK6* – which encodes a protein that acts to defend the plant against *Fusarium oxysporum* – and thereby ensures cotton fitness.**

Plants have evolved immune systems for survival and reproduction, part of immobile living and consequent exposure to a variety of stresses (Jones and Dangl, 2006). Notably, limited resources mean that such immunity often happens at the cost of growth and development and, as a result, complicated mechanisms have been developed to confine plant immune responses to certain levels in order to ensure normal development and physiology (Box 1; Huot *et al.*, 2014). Turning to crops, over time selective breeding to maximize their agricultural productivity has reduced genetic diversity, causing impaired immunity (Strange and Scott, 2005). As a result, diseases have become one of the major factors causing crop losses. However, classical strategies, including agricultural chemical control and breeding with disease-resistant wild ecotypes, are not only expensive and time-consuming but also increasingly ineffective. Molecular breeding has become one of the most promising tools to mitigate the effects of various diseases – in combination with traditional breeding methods it can increase crop resistance to various diseases and therefore greatly improve crop fitness and productivity. Thus, understanding the mechanisms governing plant immune responses and related constraints will benefit molecular breeding practices used to improve crop traits.

**Box 1. The growth/defense balance and control of plant immunity**

Plant growth and defense are required for plant fitness (left): both insufficient and excess immunity impairs fitness, resulting in yield loss in crops. A simplified model is shown (right) for the action of MAPK cascades and miRNAs in plant immunity. Plant receptors recognize pathogens and transmit the signals to the downstream MAPK cascades, which regulate growth-related signaling (auxin; BR, brassinosteroid; GA, gibberellic acid), HR-like cell death, and resistance-related signaling (JA, jasmonic acid; SA, salicylic acid; ET, ethylene). miRNA biogenesis can be positively or negatively affected by the pathogen-activated MAPK cascades and other responses triggered by pathogens at transcriptional and/or post-transcriptional levels. In turn, miRNAs can promote or inhibit plant immunity through repressing the expression of genes involved in immune responses. Arrows may indicate multiple steps; dashed lines with arrows indicate the presence of both positive and negative regulation.

**Figure:**
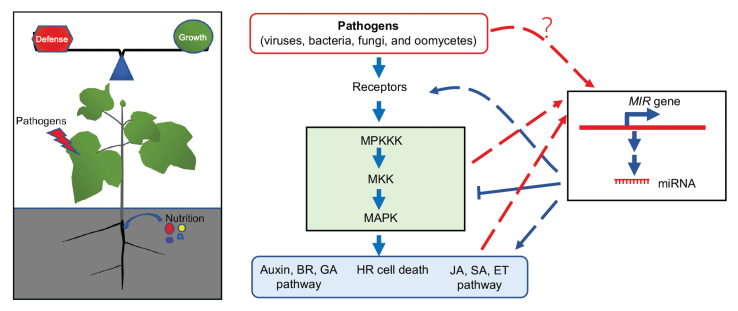

Wang *et al.* (2017) have reported that mitogen-activated protein kinase kinase 6 (MAPKK6) plays important roles in cotton (*Gossypium hirsutum*) resistance to the phytopathogenic fungus *Fusarium oxysporum* f. sp. *vasinfectum* (*F. oxysporum*), which causes one of the most serious diseases of cotton (Gaspar *et al.*, 2014). Remarkably, the authors further show that a newly identified microRNA (miRNA), ghr-miR5272a, can avoid an excessive MAPKK6-mediated immune response through repression of *MAPKK6*, thereby promoting cotton fitness (Wang *et al.*, 2017).

## The MAP kinase cascade and immune responses

In eukaryotes the conserved MAPK cascades transduce signals from upstream receptors to their downstream targets. Each MAPK cascade contains three protein kinases: MAPKKK/MEKK, MAPKK/MEK and MAPK (Xu and Zhang, 2015). Among the three kinases, MAPKKK acts upstream, perceives the signal and activates MAPKK through protein phosphorylation, which in turn phosphorylates MAPK leading to downstream gene regulation (Box 1) (Xu and Zhang, 2015). Activation of MAPK cascades is among the earliest events in plant immunity, and they play important roles in both basal immunity triggered by pathogen (microbe)-associated molecular patterns (PAMPs), which are highly conserved molecules in microorganisms, and effector-trigged immunity (ETI).

Activation of MAPK cascades regulates multiple immune responses such as the generation of reactive oxygen species (ROS), the biogenesis of the stress/defense hormones salicylic acid (SA), jasmonic acid (JA) and ethylene (ET), cell wall strengthening, and cell death caused by the hypersensitive response (HR) (Box 1) (Meng and Zhang, 2013). The MAPK cascades are also required for plant growth and development. They regulate both cell proliferation and cell differentiation and are involved in gametogenesis, embryo development, morphogenesis, senescence, abscission, fertilization and seed formation (Rodriguez *et al.*, 2010; Xu and Zhang, 2015). Notably, the same MAPK cascade can function in both defense and growth regulation (Box 1), suggesting that trade-offs are needed for the activities of MAPK cascades during plant immune responses.

MAPKK6 is one of the MAPKKs and has diverse roles in plants. In Arabidopsis, MAPKK6 targets MAPK4 and MAPK11 to regulate cytokinesis, after the MAPKKKs ANP1, ANP2 and ANP3 activate it (Kosetsu *et al.*, 2010; Zeng *et al.*, 2011). It also regulates auxin signaling through targeting MAPK12 (Lee *et al.*, 2009). In rice, MAPKK6 enhances resistance to chilling stress through activating MAPK3 (Xie *et al.*, 2012). Moreover, in tobacco, MEK1, which is a homolog of MAPKK6, activates the MAPK NTF6 to regulate resistance to tobacco mosaic virus (Liu *et al.*, 2004) and to control nitric oxide and NADPH oxidase-dependent oxidative bursts triggered by the oomycete pathogen *Phytophthora infestans* (Asai *et al.*, 2008).

Wang *et al.* have now shown that the cotton MAPKK6 displays high sequence similarity with MAPKK6 from Arabidopsis and tobacco and is expressed universally, but at highest levels in roots (Wang *et al.*, 2017). SA, methyl jasmonate (MeJA) and *F. oxyspo*rum negatively regulate the expression level of MAPKK6, suggesting that it may be involved in the cotton immune response. Moreover, knockdown of *MAPKK6* reduces cotton resistance to *F. oxysporum.* Reduction of MAPKK expression impairs induction of the SA pathway but not the JA pathway. Intriguingly, overexpression of MAPKK6 leads to the induction of both SA and JA pathways (Wang *et al.*, 2017). These data demonstrate that MAPKK6 positively regulates cotton resistance to *F. oxysporum* through promoting the SA and JA pathways. However, overexpression of MAPKK6 causes an excessive hypersensitive response (HR), resulting in lesion-mimicking phenotypes. This result raises a question as to how cotton limits the MAPKK6 activity to a certain level during *F. oxysporum* infection.

## miRNAs and plant immunity

The miRNAs, which are ~21–24 nucleotides (nt) in size, are important regulators of gene expression. They are derived from primary miRNA transcripts (pri-miRNAs), which are mainly transcribed by DNA-dependent RNA polymerase II (Pol II) (Box 1) (Zhang *et al.*, 2015). The RNase III enzyme DICER-LIKE1 (DCL1) releases the miRNAs from the stem-loop residing in pri-miRNAs. Following production, miRNAs bind and guide their effector protein, ARGONAUTE (AGO), to repress the expression of genes through cleavage of target mRNAs or translational inhibition (Chen, 2008).

miRNAs play important roles in regulating plant immunity. Some, such as miR393 and miR393*, promote immune responses, while others, including miR398 and miR773, inhibit antibacterial reactions (Navarro *et al.*, 2006; Li *et al.*, 2010; Zhang *et al.*, 2011). Interestingly, miRNAs also play roles in fine-tuning immunity reactions to a suitable level (Li *et al.*, 2017). For instance, plant NLR proteins are sensitive immune receptors, activating strong defense responses. Their levels need to be tightly controlled to avoid autoimmunity and massive loss of fitness. Many plants use miRNAs to target NLR and thus limit potential autoimmunity (Li *et al.*, 2012). More intriguingly, miRNA-mediated NLR silencing can be repressed during viral or bacterial infection, suggesting that it may be an important switch for immunity.

Despite progress in model plants, the roles of miRNAs in regulating crop immunity have been much less explored. Wang *et al.* (2017) find that a conserved miRNA, ghr-miR5272a, targets MAPKK6 and inhibits its expression through target cleavage. The expression pattern of ghr-miR5272 is reversibly correlated with that of MAPKK6 after *F. oxysporum* treatment, suggesting that ghr-miR5272a may contribute to cotton immunity through repression of MAPKK6. Indeed, overexpression of ghr-miR5272a reduces the expression of MAPKK6 and disease-resistant genes from the SA and JA pathways, causing cotton to become more susceptible to *F. oxysporum* (Wang *et al.*, 2017). This phenotype resembles that of MAPKK6 knockdown lines and reveals that ghr-miR5272a is a negative regulator of MAPKK6 during *F. oxysporum* infection. Interestingly, JA but not SA induces the expression of ghr-miR5272a (Wang *et al.*, 2017). Taken together, the data presented by Wang *et al.* provide a novel miRNA–MAPK cascade feedback loop for regulating cotton immune responses. In this loop, *F. oxysporum* infection activates the MAPKK6 cascade to trigger the expression of JA and SA pathways. The JA pathway subsequently promotes the expression of ghr-miR5272a, leading to the repression of MAPKK6 and thereby repressing the HR caused by unlimited MAPKK6 activity.

## Perspectives

Both miRNAs and MAPK cascades function as the key players in plant development and stress responses. The findings of Wang *et al*. build a link between them and advance our understanding of mechanisms balancing immunity and growth. The work also raises many questions. What are the upstream MAPKKKs and downstream MAPKs? How does JA stimulate the transcription of ghr-miR5272a? How is the balance between MAPKK6 and ghr-miR5272a reached? And is the ghr-miR5272a–MAPKK6 feedback loop a general defense mechanism against fungi or specific to *F. oxysporum*? Moreover, plants use multiple mechanisms to balance immunity and growth. It remains a challenge to understand how these mechanisms are integrated into the regulatory network, acting coordinately to ensure plant perpetuation. Finding answers will ultimately enable new breeding strategies which maximize crop fitness.
